# Pressurized Liquid Extraction Combined with Enzymatic-Assisted Extraction to Obtain Bioactive Non-Extractable Polyphenols from Sweet Cherry (*Prunus avium* L.) Pomace

**DOI:** 10.3390/nu13093242

**Published:** 2021-09-17

**Authors:** Gloria Domínguez-Rodríguez, María Concepción García, María Luisa Marina, Merichel Plaza

**Affiliations:** 1 Universidad de Alcalá, Departamento de Química Analítica, Química Física e Ingeniería Química, Facultad de Ciencias, Ctra, Madrid-Barcelona Km 33.600, 28871 Alcalá de Henares, Madrid, Spain; gloria.dominguezr@uah.es (G.D.-R.); concepcion.garcia@uah.es (M.C.G.); mluisa.marina@uah.es (M.L.M.); 2 Universidad de Alcalá, Instituto de Investigación Química Andrés M. del Río (IQAR), Ctra, Madrid-Barcelona Km 33.600, 28871 Alcalá de Henares, Madrid, Spain

**Keywords:** enzyme-assisted extraction, non-extractable polyphenols, pressurized liquid extraction, proanthocyanidins, sweet cherry pomace

## Abstract

Sweet cherry generates large amounts of by-products within which pomace can be a source of bioactive phenolic compounds. Commonly, phenolic compounds have been obtained by conventional extraction methodologies. However, a significant fraction, called non-extractable polyphenols (NEPs), stays held in the conventional extraction residues. Therefore, in the present work, the release of NEPs from cherry pomace using pressurized liquid extraction (PLE) combined with enzyme-assisted extraction (EAE) using Promod^TM^ enzyme is investigated for the first time. In order to study the influence of temperature, time, and pH on the NEPs extraction, a response surface methodology was carried out. PLE-EAE extracts displayed higher TPC (75 ± 8 mg GAE/100 g sample) as well as, PA content, and antioxidant capacity than the extracts obtained by PLE (with a TPC value of 14 ± 1 mg GAE/100 g sample) under the same extraction conditions, and those obtained by conventional methods (TPC of 8.30 ± 0.05 mg GAE/100 g sample). Thus, PLE-EAE treatment was more selective and sustainable to release NEPs from sweet cherry pomace compared with PLE without EAE treatment. Besides, size-exclusion chromatography profiles showed that PLE-EAE allowed obtaining NEPs with higher molecular weight (>8000 Da) than PLE alone.

## 1. Introduction

Phenolic compounds have been extensively investigated because their potential activity as antioxidants. Vegetables, fruits, and grains are the main sources of these compounds. The processing of these products originates a significant amount of by-products containing high quantities of phenolic compounds [1]. In particular, sweet cherries are processed into different products, such as marmalades or juices, generating important amounts of by-products among which the pomace may be highlighted as the main residue [2]. Sweet cherry pomace represents an undervalued source of bioactive phenolic compounds like flavonols, flavan-3-ols, anthocyanins, or hydroxycinnamic acids that present antioxidant, anticarcinogenic, and antihypertensive capacities, among others [3]. These types of phenolic compounds have been recovered by different extraction methodologies from food by-products to be used in the pharmaceutical, cosmetic, or food industries. Conventional extraction is the most used technique to obtain phenolic compounds, but requires a high volume of solvents and long extraction times and provides low reproducibility and selectivity [4]. In order to mitigate these problems, advanced extraction techniques such as pressurized liquid extraction (PLE) have been used to increase the efficiency and speed to extract phenolic compounds using a lower volume of solvents resulting in a more sustainable process [5]. PLE is based on the employ of solvents at high pressure and temperature maintaining the solvent in the liquid state and increasing its penetration into the food matrix to extract bioactive compounds [6]. However, conventional and advanced extraction techniques do not allow to extract an important fraction of bioactive phenolic compounds called non-extractable polyphenols (NEPs). NEPs have been shown to possess high antioxidant, antihypertensive, and antidiabetic properties [7,8,9]. NEPs comprise different classes of phenolic compounds such as phenolics with high molecular weight or simple phenols associated with macromolecules, like proteins, in the cell wall [10]. These compounds remain in the residue obtained by conventional extraction due to their strong interactions with the cell wall. 

Recovery of bioactive NEPs requires an additional treatment to break these interactions. In this sense, acid and alkaline hydrolysis are the most used treatments to release NEPs from the residue of conventional extractions [9]. Nevertheless, these hydrolysis treatments are non-specific and use aggressive pH values that might change the conformation of NEPs [11]. Thus, enzymatic-assisted extraction has emerged in the last years as one of the most selective and environmentally sustainable techniques to extract NEPs. Different enzymes have been employed to release NEPs from food matrices such as pectinases, cellulases, tannases, and glucuronidases, among others [12,13]. In the case of NEPs from sweet cherry pomace, EAE has proven to extract bioactive NEPs with higher molecular weight than acid hydrolysis. In fact, extracts obtained by EAE from sweet cherry pomace presented higher proanthocyanidin content and antioxidant capacity than the extracts obtained by acid and alkaline hydrolysis [9]. However, as far as we know, there are no studies based on the combination of PLE with EAE to release NEPs from the matrix. For instance, EAE and PLE were compared when used separately to extract bioactive compounds from lemon balm observing that extracts obtained by PLE presented higher bioactive phenolic compounds than EAE [14]. Besides, PLE has been used after EAE with the extraction residue of EAE to obtain phenolic compounds from *Sargassum muticum* seaweed, but the studies about the combination of both extraction techniques are very limited [15]. 

For this reason, this work aimed to increase the efficiency in the extraction of antioxidant NEPs from the residue of conventional extraction of sweet cherry pomace (*Prunus avium* L.) developing an optimum extraction method based on the combination of PLE with EAE. Promod^TM^ enzyme with protease activity was selected because it was the most appropriate enzyme, based on previous studies, to extract NEPs from sweet cherry pomace [9]. A Box-Behnken experimental design was used to determine the influence of time, temperature, and solvent pH on the extraction conditions to obtain high phenolic and proanthocyanidin contents with high antioxidant capacity. Additionally, extracts performed under the optimal extraction conditions to obtain antioxidant NEPs by PLE-EAE were compared with extracts obtained using the same conditions with PLE without EAE and with conventional extraction. Furthermore, a determination of the distribution of the molecular weight of NEPs extracted by PLE-EAE and PLE was carried out by HPLC-SEC.

## 2. Materials and Methods

### 2.1. Chemicals and Reagents

Ethanol, hydrochloric acid (37%), and acetone of HPLC grade were purchased from Scharlab Chemie (Barcelona, Spain). Gallic acid, Folin-Ciocalteu reagent, epicatechin, vanillin, polyethylene glycol (8000 Da), polyethylene glycol (4000 Da), twin20 (1228 Da), ethylene glycol (62 Da), dextran (50,000 Da), iron(III) chloride, sodium carbonate, hydrogen peroxide, 4-dimethylaminocinnamaldehyde (DMAC), 6-hydroxy-2,5,7,8-tetramethyl-chromane-2-carboxylic acid (Trolox), potassium persulfate, 2,2’-azinobis(3-ethylbenzothiazoline-6-sulphonic acid) and diammonium salt (ABTS)were purchased from Sigma-Aldrich (Saint Louis, MO, USA). Sodium dihydrogen phosphate dihydrate and dipotassium hydrogen phosphate were obtained from Merck (Hesse, Darmstadt, Germany). 

Methanol (99.99%), formic acid, acetonitrile, and butanol of HPLC grade were supplied by Fisher Scientific (Midlands, Leicestershire, UK). Ultrapure water (18.2 MΩ/cm) was obtained from a Millipore system (Millipore, Billerica, MA, USA). Promod 439 L^TM^ enzyme was a kind donation of Biocatalysts Ltd. (Wales, Cardiff, UK).

### 2.2. Samples

Sweet cherries from the *Prunus avium* L. genus, Early Lory variety, and Rosaceae family were selected from La Almunia de Doña Godina (Zaragoza, Spain) for this study. Fruits were washed, de-stemmed, de-stoned, and pressed manually to attain the pomace. Finally, the pomace was stored at −20 °C until its analysis.

### 2.3. Conventional Extraction of Extractable Polyphenols (EPPs)

Extractable polyphenols were obtained from the cherry pomace through the method employed by Condezo-Hoyos et al. [16] with some modifications [9]. Twenty mL of methanol/water (50:50, *v*/*v*) acidified to pH 2 with 2 N of HCl were added to 15 g of cherry pomace and the mixture was shaken for 1 h at room temperature. To obtain the supernatant, the extract was centrifuged at 2100× *g* for 10 min. Then, extraction residue was re-extracted employing 20 mL of acetone/water (70:30, *v*/*v*) for 1 h at room temperature with shaking, followed by centrifugation at 2100× *g* for 10 min. Subsequently, methanol and acetone supernatants were mixed. Extract and extraction residue were stored at −20 °C until the analysis of extractable polyphenols and the NEPs extraction, respectively. The extraction was carried out in triplicate.

### 2.4. Release of Non-Extractable Polyphenols (NEPs)

An experimental design was carried out using PLE combined with EAE employing Promod 439 L^TM^ enzyme to optimize the NEPs extraction from the residue of conventional extraction of sweet cherry pomace. The Box-Behnken design was used as a second-order design with three levels and five central points. To investigate the effect of time (5–40 min), temperature (60–80 °C), and pH (6–10) on the NEPs extraction, MODDE 10.1 software (Sartorius Stedim Biotech, Malmö, Sweden) was used. A Dionex ASE 150 instrument (Thermo Fisher; Baviera, Germering, Germany) was used for the extractions. Extractions were achieved in 10 mL extraction cells, which were filled with 5.5 g of the dried residue of the conventional extraction of sweet cherry pomace on were added 140 µL of Promod^TM^ enzyme/g of sample. Enzyme concentration as well as buffer phosphate (100 mM) as extraction solvent were selected for releasing NEPs from cherry pomace based on the results displayed in a previous study carried out by our research team [9]. HCl or NaOH were employed to adjust the pH of the buffer. This extraction solvent was sonicated for 30 min to remove dissolved oxygen. The cell was heated up for 6 min before each analysis.

In total, 17 experiments were carried out in a random run order (Table S1). Total phenolic content (Folin-Ciocalteu assay), total proanthocyanidin content (DMAC, vanillin, and butanol/HCl assays), and antioxidant capacity (Trolox Equivalent Antioxidant Capacity or TEAC) and inhibition of hydroxyl radical assays) were selected as response variables.

The adequacy of fitted models between time, temperature, and pH and response variables was evaluated by analysis of variance (ANOVA). Graphical and numerical analyses based on the response surface plots and the criteria of the desirability function were employed to calculate the theoretical optimal processing conditions. Finally, experimental extractions were carried out using the theoretical optimal extraction conditions to verify the study. Additionally, PLE without enzyme was also performed under the optimal extraction conditions achieved by the Box-Behnken experimental design.

### 2.5. Total Phenolic Content (TPC)

A Folin-Ciocalteu (FC) assay was employed to determine TPC following the method described by Kosar et al. [17] with some modifications. Briefly, 600 µL of water, 50 µL of undiluted FC reagent, and 10 µL of sample were mixed under shaken. After 1 min, 150 µL of 2% (*w*/*v*) Na_2_CO_3_ and 190 µL of water were added to the mixture and were shaking. After completing the reaction at 20 °C for 2 h, the absorbance was measured at 760 nm in a Cary 8454 UV-Vis spectrophotometer (Agilent Technologies, Palo Alto, CA, USA). Finally, results were expressed as mg of gallic acid equivalents (GAE)/100 g sample. 

### 2.6. Total Proanthocyanidin Content

#### 2.6.1. DMAC Assay

Total proanthocyanidin content (PA) was measured through DMAC method used by Montero et al. [18]. In order to prepare the DMAC solution, 0.1% DMAC reagent (*w*/*v*) was used on ethanol/water/HCl (75:12.5:12.5, *v*/*v*/*v*). The extract (140 µL) was mixed with 420 µL of DMAC solution and after 15 min at room temperature, the absorbance was read at 640 nm. Blanks with 140 µL of methanol instead of sample and control samples without DMAC solution were included. Results were expressed as mg of epicatechin/100 g sample. 

#### 2.6.2. Vanillin Assay

Vanillin assay was employed according to Gu et al. [19] to measure the total PA content. Briefly, 1.7 mL of a solution of 0.5% vanillin and 4% concentrated HCl in methanol was mixed with 100 µL of extract. After 20 min at room temperature, the absorbance was read at 500 nm. The amount of PAs was expressed as mg epicatechin/100 g sample.

#### 2.6.3. HCl/Butanol Assay

HCl/butanol assay described by Pérez-Jiménez et al. [20] with some modifications was applied. Briefly, 200 µL of extract were added to 800 µL of HCl/butanol (5:95, *v*/*v*). They were let to react for 1 h at 100 °C. Then, tubes were centrifuged at 2500× *g* for 10 min and the supernatants were collected. Subsequently, the absorbance was measured at 555 nm and the results were expressed as mg epicatechin/100 g sample. 

### 2.7. Antioxidant Capacity Determination

#### 2.7.1. Trolox Equivalent Antioxidant Capacity (TEAC) Assay

TEAC assay was utilized following the method described by Re et al. [21]. To form the ABTS radical cation (ABTS^∙+^), a stock solution of 7 mM ABTS was made to react with 2.45 mM potassium persulfate during 12–16 h at room temperature and under darkness. The stock solution was diluted with 5 mM phosphate buffer (pH 7.4) to form the work solution until absorbance reached values of 0.70 (±0.02) AU at 734 nm. The reaction was started by adding 10 µL of different sample concentrations to 990 µL of work solution. The bleaching of ABTS was followed at 734 nm at room temperature until completely reacted (45 min). Trolox was used as a reference standard to express the results as TEAC values (µmol Trolox/g sample) employing four different concentrations of each extract giving a linear response between 20 and 80% comparing with the initial absorbance. 

#### 2.7.2. Capacity to Inhibit the Formation of Hydroxyl Radical Assay

The capacity to inhibit the formation of hydroxyl radicals was measured using the Ajibola et al. [22] method. Fifty µL of a 3 mM solution of 1,10 phenanthroline in 0.1 M of phosphate buffer (pH 7.4) was added to 50 µL of 3 mM FeSO_4_ in water, 50 µL of sample, and 50 µL of 0.01% H_2_O_2_. Then, the mixture was incubated for 1 h at 37 °C and 700 rpm in a Thermomixer Compact (Eppendorf AG, Hamburg, Germany). The capacity to inhibit the formation of hydroxyl radicals was obtained by measuring the absorbance at 536 nm. The % of hydroxyl radical formation inhibition was expressed through the following equation:

$$\%=\frac{Abs\;sample-Abs\;blank}{Abs\;control-Abs\;blank}\times 100$$

where *Abs sample* is the absorbance of the sample, *Abs blank* is the absorbance of the buffer and *Abs control* is the absorbance of the solution prepared with water instead of H_2_O_2_.

### 2.8. High-Performance Liquid Size-Exclusion Chromatography (HPLC-SEC) Determination of Molecular Weight of NEPs from Sweet Cherry Pomace Extracts

To determine the phenolic profile of NEPs obtained by PLE with Promod^TM^ enzyme and PLE without enzyme under the optimal extraction conditions, size-exclusion chromatography (SEC) was employed an 1100 HPLC-DAD system from Agilent (Agilent Technologies, Santa Clara, CA, USA). Separation was carried out on a SEC column (PolySep-GFC-P2000, 300 × 7.8 mm, Phenomenex, Torrance, CA, USA) with a fractionation range of 100 Da-10 KDa. Separation conditions were applied according to Domínguez-Rodríguez et al. [9], where 100% water was used in isocratic mode at 0.3 mL/min as mobile phase for 60 min with a column temperature of 25 °C and an injection volume of 20 µL. Twenty microliters of extract were injected. The detection wavelength employed was 280 nm. In order to calibrate the molecular weight of the SEC column, polyethylene glycol (8000 Da), polyethylene glycol (4000 Da), twin20 (1228 Da), and ethylene glycol (62 Da) were employed as standards. The calibration curve obtained plotting molecular weight (MW) as a function of retention time (min) was employed to determine the MW of the extracted NEPs. Linear equation (y = −0.0028x + 37.043) with an R^2^ determination coefficient value of 0.9122 was used to express the responses obtained. The void volume was determined with dextran (50,000 Da).

### 2.9. Statistical Analysis

Statistical software Statgraphics Centurion version XVII (Statistical Graphics Corp, The Plains, VA, USA) was used to observe differences in TPC, PA contents, and antioxidant capacity between PLE with Promod^TM^ enzyme extracts, PLE without enzyme extracts, and conventional extraction extracts. ANOVA by Fisher’s exact test allowed to determine statistically significant differences (*p* ≤ 0.05) between mean values for different extracts at 95% confidence level. All the analyses were carried out in triplicate for each extract. All the analyses were carried out in triplicate for each extraction.

## 3. Results and Discussion

This work describes for the first time the use and optimization of the combination of two eco-friendly extraction methodologies based on PLE with EAE to release NEPs from the residue of conventional extraction of sweet cherry pomace. 

### 3.1. Optimization of NEPs Extraction from Cherry Pomace Extraction Residue by PLE Combined with EAE

The extraction of NEPs was carried out from the residue obtained by the conventional extraction of polyphenols from sweet cherry pomace. Promod^TM^ enzyme was selected because it was the most efficient in the extraction of bioactive NEPs from cherry pomace compared with Pectinase^TM^ and Depol^TM^ enzymes in a previous work performed by our research group [9]. Other conditions consisted of the use of a phosphate buffer (100 mM) and an enzyme concentration of 140 µL of Promod^TM^ enzyme/g of sample. This enzyme was added to the residue of conventional extraction before starting the PLE process. Promod^TM^ enzyme presents protease and polygalacturonase activities and allows to modify the functionality of the proteins as well as to solubilize proteins and their aggregates improving the release of NEPs from the cell wall of the matrix and aggregates. The protease activity enables to break the proteic tonoplast surrounding cellular vacuoles contributing to the release of phenolic compounds contained in them. On the other hand, the polygalacturonase activity enables to disrupt α-1,4-glycosidic bonds on polygalacturonic acid of pectins degrading the pectic chain and releasing phenolic compounds that interact with carboxyl and hydroxyl groups of pectin [23,24].

Once the enzyme was added, extraction processes were carried out according to Box Behnken experimental design to optimize the influence of time (5–40 min), temperature (60–80 °C), and solvent pH (6–10) on six response variables (FC, DMAC, vanillin, butanol/HCl, TEAC, and hydroxyl radical assays). The time, temperature, and pH ranges to carry out the experimental design were established taken into account the results obtained by Domínguez-Rodríguez et al. [9] and according to the enzyme specifications. The 17 experiments established by Box Behnken design with their respective TPC, total PA content, and antioxidant capacity values are summarized in Table S1. Table 1 shows the analysis of variance, goodness of fit, and the adequacy of the model. For instance, it can be observed that the regression of the model of Promod^TM^ enzyme could explain a satisfactory developed model with a range from 81.6 to 97.1% of the results obtained by FC, DMAC, vanillin, butanol/HCl, TEAC, and hydroxyl radical assays. In addition, the standard error of the regression model expressed as relative standard deviation (RSD) was below 6.01% in all assays. Besides, ANOVA analysis showed an adequate regression model for the responses of vanillin, butanol/HCl, TEAC, and inhibition of hydroxyl radical assays since it presented a *p*-value for the regression lower than 0.05. However, the regression model was not adequate for FC and DMAC assays since they showed *p*-values of 0.073 and 0.167, respectively. Additionally, all responses displayed an adequate *p*-value for the lack-of-fit test, presenting values higher than 0.05 (Table 1). 

**Figure 1:**
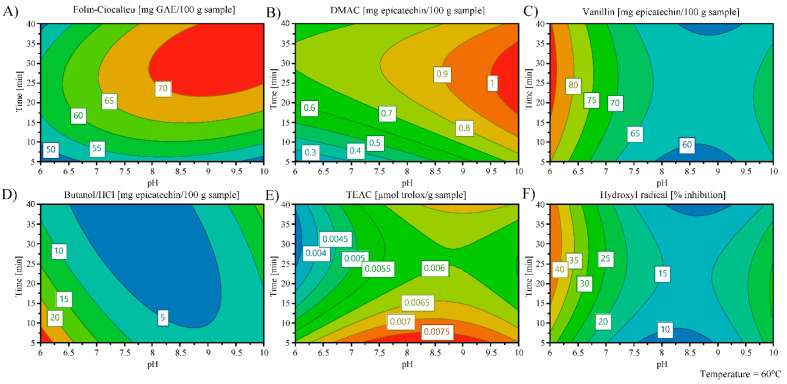
Contour plots presenting the effect of time (min) and pH at the optimum extraction temperature (60 °C) on the TPC (Folin-Ciocalteu method, mg GAE/g sample (**A**)), total PA content (DMAC (**B**), vanillin (**C**), and butanol/HCl (**D**) assays, mg epicatechin/100 g sample), and total antioxidant capacity (TEAC (µmol Trolox/g sample) (**E**) and capacity to inhibit the formation of hydroxyl radical (% inhibition) (**F**) methods) from PLE-EAE extracts.

Furthermore, the ANOVA test was used to determine the main variables (time, temperature, and/or pH) that can affect the response factors with a significant effect (*p*-value < 0.05) (see Table 1). The time followed by the temperature were the variables that presented less significant effects on the different responses. That is why, as can be seen in Figure 1, to observe the effects of variables (time and pH) on the five responses, the counterplots were fixed at the optimum extraction temperature for obtaining the highest response values (60 °C). The TPC values increased at high pH (*p*-value < 0.05) while the temperature and time did not have a significant effect (*p*-value > 0.05) (Figure 1A and Table 1). The extraction time, temperature, and pH did not present a significant effect on the extraction of PAs when these compounds were measured by DMAC and vanillin assays (*p*-value > 0.05) (Figure 1B,C and Table 1). However, when the PA content was determined by butanol/HCl assay, the pH presented a negative effect on their extraction (*p*-value < 0.05). Therefore, when a high pH was employed in the extraction of PAs, the PA content decreased (Figure 1D). Concerning antioxidant capacity, the time presented a negative effect while temperature and pH showed a positive impact on the TEAC value (see Table 1). In this sense, Figure 1E shows that the antioxidant capacity measured by TEAC assay was higher at lower extraction times but at higher temperatures and pH values (*p*-value < 0.05). As can be seen in Table 1, the positive and negative effects of temperature and pH, respectively, were observed on antioxidant capacity measured by the inhibition of hydroxyl radical assay. In fact, Figure 1F shows that high temperatures and low pH allowed to obtain extracts with a high capacity to inhibit the formation of hydroxyl radicals.

Therefore, 60 °C for 31 min at pH 10 were the optimal extraction conditions to obtain the highest TPC, PA content, and antioxidant capacity from cherry pomace extraction residue. These optimal extraction conditions were employed to release antioxidant NEPs from cherry pomace extraction residue. Table 2 shows the predicted theoretical values obtained under the optimal extraction conditions of TPC, total PA content (DMAC, vanillin, and butanol/HCl assays), and total antioxidant capacity (TEAC and percentage of inhibition of hydroxyl radical formation assays). As can be seen in Table 2, experimental values of TPC, PA content, and antioxidant capacity were within the range of theoretical values obtained by Box-Behnken design and closer to optimum value excepting the total PA content values obtained by vanillin and butanol/HCl assays which were closer to the upper theoretical value. This means that the predictive model from experimental design allowed to attain a good prediction to release antioxidant NEPs from cherry pomace extraction residue by PLE in combination with EAE with Promod^TM^ enzyme.

### 3.2. Comparison of PLE Combined with EAE and PLE Alone to Release NEPs from Cherry Pomace Extraction Residue

The efficiency of the combination of both advanced extraction techniques, PLE and EAE with Promod^TM^ enzyme, for the recovery of NEPs from the extraction residue of sweet cherry pomace (obtained after the recovery of EPPs) was next evaluated. For that purpose, PLE without EAE was carried out under the optimal extraction conditions achieved by Box-Behnken experimental design (60 °C for 31 min at pH 10) and results were compared to the obtained when using simultaneously PLE and EAE.

**Figure 2:**
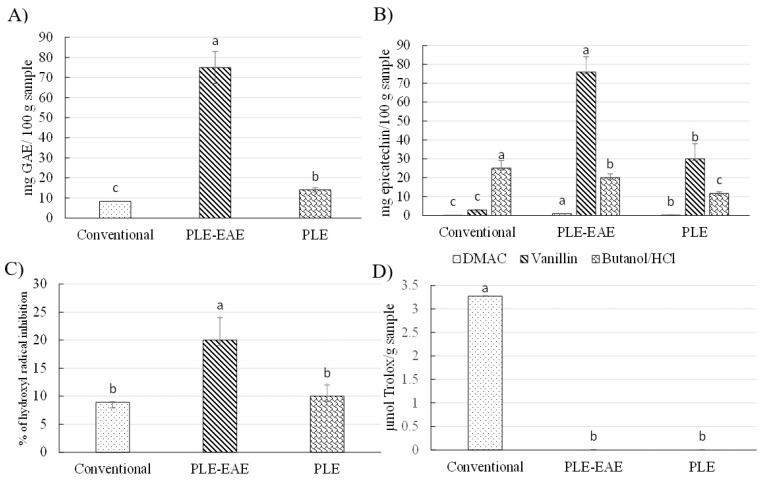
Experimental values of (**A**) TPC (Folin-Ciocalteu method), (**B**) total PA content (DMAC, vanillin, and butanol/HCl assays), and antioxidant capacity by (**C**) inhibition of hydroxyl radical and (**D**) TEAC assays obtained under the optimal PLE-EAE conditions and PLE without enzyme from the extraction residue of sweet cherry pomace and EPPs conventional extraction from sweet cherry pomace. ^a,b,c^ Letters show the significant differences among extraction methods of NEPs (*p* ≤ 0.05).

As can be seen in Figure 2, results obtained for the EPPs extract, and NEPs extract by PLE with and without enzyme showed statistically significant differences. As can be observed, EPPs extract presented the lowest TPC and PA content values obtained by Folin-Ciocalteu, DMAC, and vanillin assays. However, PA content using butanol/HCl assay was higher in EPPs extract than in PLE-EAE and PLE extracts (Figure 2B). This fact may be because each assay has a different reaction mechanism since butanol/HCl is more specific to determine polymeric polyphenols while DMAC and vanillin assay react with monomeric polyphenols [9]. 

On the other hand, TPC, PA content obtained by vanillin, DMAC, and butanol/HCl assays and antioxidant capacity measured by TEAC and percentage of inhibition of hydroxyl radical assays showed higher values in PLE combined with EAE with Promod^TM^ enzyme extract than PLE without EAE extracts. 

Despite PLE is an advanced extraction methodology that increases the extraction yields and reproducibility compared with conventional extraction techniques, this extraction methodology alone produced an incomplete extraction of NEPs. Possibly, this inefficient extraction could be due to the strong interactions of NEPs with the matrix. Different compounds of the food matrix can interact with NEPs being inaccessible to pressurized liquids or conventional extraction techniques [25]. However, the combination of PLE with EAE allowed releasing higher PA content than extracts obtained with PLE without EAE. In this sense, the protease and polygalacturonase activity of Promod^TM^ enzyme could have released NEPs from the cell wall of the residue of the extraction of sweet cherry pomace. In fact, protease activity promotes the breakage of ester linkages with carboxylic groups in proteins and polygalacturonase activity makes possible the disruption of hydroxyl groups of pectins [26,27]. The overall effect is the increase in the release of NEPs. 

Additionally, PLE-EAE and PLE extracts presented lower TPC values than TPC values obtained by Domínguez-Rodríguez et al. [9] from alkaline, acid and EAE with Promod^TM^, Depol^TM^ and Pectinase^TM^ enzymes extracts from the same variety of sweet cherry pomace. By contrast, PLE-EAE and PLE extracts showed higher polymeric non-extractable PA content than acid and alkaline hydrolysis [9]. These results indicated that the differences in TPC and PA contents between these studies may be due to the influence of climatic differences in each vintage.

Additionally, antioxidant capacity results measured by the percentage of inhibition of hydroxyl radical assay showed that the EPPs presented lower antioxidant capacity than the NEPs extracts attained by PLE-EAE (see Figure 2C). Nevertheless, TEAC values for EPPs extracts showed higher antioxidant capacity (3.27 µmol Trolox/g sample) than PLE-EAE and PLE extracts (Figure 2D). The differences between antioxidant methods may be due to the different reaction mechanisms of both antioxidant assays. In fact, in TEAC assay a radical (ABTS) is employed that is not found in food or biological systems [28,29,30] while in the percentage of inhibition of hydroxyl radical assay one of the most relevant radicals generated in our body is used and, thus, the percentage of inhibition of hydroxyl radical assay could provide an approximation of the antioxidant effect of the extracts on the organism. 

PLE-EAE and PLE extracts presented higher non-extractable PA content but lower antioxidant capacity than the extracts obtained by Domínguez-Rodríguez et al. [9] from EAE with Promod^TM^ enzyme without PLE from the same variety of sweet cherry pomace [9]. However, these results cannot be strictly compared because the sweet cherry pomace from this work and the previous one correspond to different vintages and the initial composition of phenolic compounds was different in both pomaces. PLE displayed a positive effect on the extraction of NEPs since in combination with EAE showed higher antioxidant capacity than PLE alone in TEAC and inhibition of hydroxyl radical assays. As can be observed, different results were obtained depending on the extraction method and analytical assay employed due to different compounds are extracted and interact depending on the reaction mechanism of each assay. In general, results showed that NEPs remain retained in the residue of the conventional extraction of sweet cherry pomace and the combination of PLE-EAE enhances the access to release these compounds from the food matrix.

### 3.3. Determination of Molecular Weight of NEPs by Size Exclusion Chromatography

**Figure 3:**
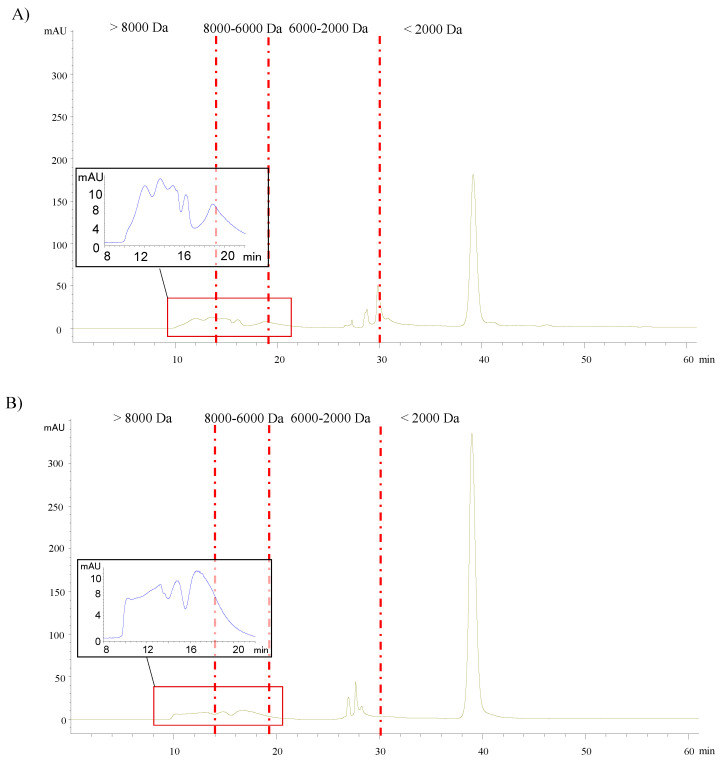
HPLC-SEC chromatograms of the extracts achieved by (**A**) PLE-EAE with Promod^TM^ enzyme and (**B**) PLE without enzyme from sweet cherry pomace extraction residue.

In order to have an estimation of the molecular weight of NEPs present in PLE-EAE and PLE extracts from sweet cherry pomace residue (obtained after EPPs conventional extraction), HPLC-SEC analysis was carried out. As can be observed in Figure 3, the chromatographic analysis showed higher signal intensity in the extracts obtained only by PLE than in that from PLE-EAE. 

The molecular weight distribution in the main peaks of NEPs extracted with <2000 Da and 2000–6000 Da was similar in both chromatograms obtained from the PLE-EAE and PLE extracts, notwithstanding low molecular weight compounds, were more abundant in PLE than PLE-EAE extracts (Figure 3). However, PLE-EAE presented higher peak areas which corresponded to high molecular weight compounds. In fact, PLE-EAE extract exhibited a higher area of compounds with a molecular weight from 2000 Da to >8000 Da than PLE extract highlighting compounds with a molecular weight > 8000 Da that showed an area of 2145 ± 70 in PLE-EAE extract (see Table 3).

By contrast, PLE extract presented a higher area of compounds with a molecular weight lower than 2000 Da than PLE-EAE extract. Compounds with this molecular weight were predominant in both extracts obtained by PLE and PLE-EAE with an area of 15,580 ± 284 and 8219 ± 49, respectively (Table 3). 

In this study, PLE-EAE allowed to recover extracts with higher total peak area for NEPs than conventional extraction and acid hydrolysis from sweet cherry pomace carried out in a previous work by our research group [9], although, as it was commented above, samples analyzed proceeded from different vintages and, therefore, results are not comparable. Despite the molecular weight distribution was similar between extracts, PLE-EAE extracts showed higher total phenolic and proanthocyanidin content as well as higher antioxidant capacity than PLE extracts. For this reason, results suggested that NEPs with higher molecular weight provided higher bioactivity to the extracts.

In general, this work proposes for the first time an efficient extraction method based on the combination of PLE with EAE using Promod^TM^ enzyme with protease and polygalacturonase activities to achieve the extraction of NEPs from sweet cherry pomace.

## 4. Conclusions

In conclusion, the optimal extraction conditions to obtain NEPs using PLE-EAE with Promod^TM^ enzyme were a temperature of 60 °C for 31 min at pH 10.0. In addition, PLE-EAE extracts were compared in terms of TPC, PA content, and antioxidant capacity with PLE extracts obtained under the same optimal extraction conditions but without the addition of enzymes, and with EPPs extracts. In general, PLE-EAE was the most efficient extraction technique to release NEPs from sweet cherry pomace. This extract presented higher TPC, PA content, and antioxidant capacity than conventional and PLE extracts excepting polymeric PA content measured by butanol/HCl assay and antioxidant capacity measured by TEAC assay which presented higher values in the conventional EPPs extract. The estimation of the molecular weight distribution showed that PLE-EAE presented higher peak area of compounds with high molecular weight than PLE extract. For all these reasons, PLE-EAE can be recommended as an environmentally sustainable method to release antioxidant NEPs from sweet cherry pomace.

## Figures and Tables

**Table 1:** Coefficients of the multiple linear regression model from the experimental design for PLE combined with EAE with Promod enzyme that the best fitted responses (Folin-Ciocalteu, DMAC, vanillin, butanol/HCl, TEAC, and inhibition of hydroxyl radical assays) with the extraction parameters (t: time, T: temperature and p: pH) and the analysis of variance (ANOVA).

Parameters	Folin-Ciocalteu	*p*-Value	DMAC	*p*-Value	Vanillin	*p*-Value	Butanol/HCl	*p*-Value	TEAC	*p*-Value	Hydroxyl Radical	*p*-Value
Constant	76.5687		0.714975		50.4326		2.31323		0.00631004		12.5415	
t	3.74087	0.0690312	0.0988549	0.0757302	1.48121	0.274243	−1.55757	0.259711	−0.00057974	0.023165	−0.226677	0.874268
T	−0.642173	0.708234	−0.0983441	0.0768368	−2.6666	0.0781056	−1.7044	0.244879	0.0008498	0.0122791	3.93067	0.0248276
p	4.92316	0.0288109	0.0657091	0.197594	−0.972796	0.432818	−5.1847	0.0143429	0.00068073	0.0136543	−7.98952	0.00067382
t^2^	−5.30567	0.0321596	−0.12659	0.0499341	−1.84528	0.176053	1.47379	0.383005	0.00066431	0.0270325	−2.92402	0.0664017
T^2^	−4.74775	0.0463558	−0.020252	0.697806	4.16827	0.0162499	−0.925015	0.517185	0.00037737	0.0519364	4.04103	0.019886
p^2^	−3.79391	0.0892811	0.0230183	0.659731	7.39256	0.00278374	5.73637	0.0116925	−0.00072299	0.0206362	9.85783	0.00015956
t*T	−0.902904	0.624473	−0.0188704	0.70636	0.305061	0.84502	0.874684	0.510832	9.47 × 10^−5^	0.653919	−0.755402	0.601397
t*p	2.94867	0.149462	−0.0406045	0.429809	−0.903755	0.458469	3.93605	0.0315481	0.00025249	0.140774	−2.08563	0.174755
T*p	0.182355	0.920256	−0.0668348	0.216721	4.09835	0.0148862	−1.64187	0.361947	−8.93 × 10^−5^	0.672088	−1.09081	0.455551
R^2^	0.876		0.816		0.943		0.931		0.971		0.941	
RSD	6.063		0.1655		4.232		4.237		0.000517		5.524	
*p*-value (test of regression)	0.073		0.167		0.012		0.05		0.009		0.002	
*p*-value (lack of fit)	0.65		0.346		0.946		0.109		0.213		0.492	

**Table 2:** Theoretical and experimental values of TPC (Folin-Ciocalteu method), total PA content (DMAC, vanillin, and butanol/HCl assays), and antioxidant capacity (DPPH and TEAC methods) obtained under the optimal PLE-EAE conditions as well as experimental values obtained by PLE under the optimal PLE-EAE conditions but without enzyme. ^a,b^ Letters show the significant differences among extraction methods of NEPs (*p* ≤ 0.05).

Optimal EAE Conditions	Theoretical Values			Experimental Values	
	Optimum Value	Lower	Upper	PLE with Promod^TM^ Enzyme	PLE without Enzyme
TPC (mg GAE/100 g sample)	72.1	57.7	86.5	75 ± 8 ^a^	14 ± 1 ^b^
DMAC (mg epicatechin/100 g sample)	1.04	0.65	1.44	0.97 ± 0.07 ^a^	0.24 ± 0.03 ^b^
Vanillin (mg epicatechin/100 g sample)	66.2	53.8	78.6	76 ± 8 ^a^	30 ± 8 ^b^
Butanol/HCl (mg epicatechin/100 g sample)	13.2	2.4	24.2	20 ± 2 ^a^	11.6 ± 0.9 ^b^
TEAC (µmol Trolox/g sample)	0.0056	0.0039	0.0074	0.0051 ± 0.0006 ^a^	0.0027 ± 0.0008 ^b^
Hydroxyl radical assay (% of hydroxyl radical inhibition)	22.8	10.8	34.9	20 ± 4 ^a^	10 ± 2 ^b^

**Table 3:** Estimation of the molecular weight distribution by HPLC-SEC at 280 nm (expressed as peak area) of NEPs obtained by PLE-EAE and PLE from extraction residues of sweet cherry pomace.

Extraction Method	>8000 Da	8000–6000 Da	6000–2000 Da	<2000 Da
PLE with Promod^TM^ enzyme	2145 ± 70	1604 ± 183	1760 ± 38	8219 ± 49
PLE without enzyme	811 ± 400	1376 ± 223	569 ± 49	15,580 ± 284

## Supplementary Materials

The following are available online at https://www.mdpi.com/article/10.3390/nu13093242/s1, Table S1: Experimental design obtained by Box-Behnken and experimental results obtained under the designed conditions by PLE combined with EAE with Promod^TM^ enzyme from extraction residue of conventional extraction of sweet cherry pomace using Folin-Ciocalteu (mg GAE/100 g sample), DMAC (mg epicatechin/100 g sample), vanillin (mg epicatechin/100 g sample), butanol/HCl (mg epicatechin/100 g sample), TEAC (µmol Trolox/g sample) and the capacity to inhibit the formation of hydroxyl radical (% of hydroxyl radical inhibition) assays response factors.
