# Supplementary material for: Pressurized Liquid Extraction Combined with Enzymatic-Assisted Extraction to Obtain Bioactive Non-Extractable Polyphenols from Sweet Cherry (Prunus avium L.) Pomace
Source: Nutrients. 2021 Sep 17;13(9):3242. doi: 10.3390/nu13093242 (PMC8465171; doi:10.3390/nu13093242)
Supplement: Supplementary file 1 [file nutrients-13-03242-s001.zip › nutrients-1336213-supplementary.pdf]

nutrients

MDPI

Article

Pressurized liquid extraction combined with enzymatic-assisted extraction to obtain bioactive non-extractable poly-phenols from sweet cherry (Prunus avium L.) pomace

Gloria Domínguez-Rodríguez [1], María Concepción García [1,2], María Luisa Marina [1,2], and Merichel Plaza [1,2,*]

[1] Universidad de Alcalá, Departamento de Química Analítica e Ingeniería Química, Facultad de Ciencias, Ctra. Madrid-Barcelona Km 33.600, 28871 Alcalá de Henares (Madrid), Spain; gloria.dominguezr@uah.es (G D.-R.); concepcion.garcia@uah.es (C.G); mluisa.marina@uah.es (M.L.M); merichel.plaza@uah.es (M.P.).

[2] Universidad de Alcalá, Instituto de Investigación Química Andrés M. del Río (IQAR), Ctra. Madrid-Barcelona Km 33.600, 28871 Alcalá de Henares (Madrid), Spain

* Correspondence: merichel.plaza@uah.es; Tel.: +34-91-885-6392

nutrients

MDPI

Table S1. Experimental design obtained by Box-Behnken and experimental results obtained under the designed conditions by PLE combined with EAE with Promod enzyme from extraction residue of conventional extraction of sweet cherry pomace using Folin-Ciocalteu (mg GAE/100 g sample), DMAC (mg epicatechin/100 g sample), vanillin (mg epicatechin/100 g sample), butanol/HCl (mg epicatechin/100 g sample), TEAC (µmol Trolox/g sample) and the capacity to inhibit the formation of hydroxyl radical (% of hydroxyl radical inhibition) assays response factors.

Samples		Extraction conditions			Analytical results					
**Exp No**	**Run order**	**Time (min)**	**Temperature (ºC)**	**pH**	**Folin-Ciocalteu (mg GAE/100 g sample)**	**DMAC (mg epicat/100 g sample)**	**Vanillin (mg epicat/100 g sample)**	**Butanol/HCl (mg epicat/100 g sample)**	**TEAC (µmol Trolox/g sample)**	**Hydroxyl radical assay (% inhibition hydroxyl radical)**
**1**	15	5	60	8	52.01	0.43	60.14	7.57	0.007	7.62
**2**	4	40	60	8	70.54	0.84	112.25	0.86	0.012	14.99
**3**	6	5	80	8	50.51	0.14	47.94	1.00	0.010	17.52
**4**	10	40	80	8	62.73	0.42	52.25	0.51	0.010	18.87
**5**	9	5	70	6	58.40	0.38	58.70	30.70	0.007	37.10
**6**	17	40	70	6	52.50	0.70	65.20	15.36	0.004	39.77
**7**	5	5	70	10	58.48	0.51	58.04	1.22	0.007	21.37
**8**	16	40	70	10	73.21	0.54	57.65	12.61	0.007	7.42
**9**	11	22.5	60	6	53.51	0.50	85.95	14.83	0.004	41.07
**10**	13	22.5	80	6	54.13	0.58	61.81	12.62	0.006	60.68
**11**	1	22.5	60	10	68.51	1.10	67.30	15.05	0.006	24.43
**12**	8	22.5	80	10	70.41	0.70	74.22	36.26	0.014	35.34
**13**	3	22.5	70	8	82.60	0.74	53.56	3.98	0.006	13.23
**14**	12	22.5	70	8	69.20	0.42	51.43	13.40	0.006	14.02
**15**	2	22.5	70	8	57.41	0.83	55.60	0.48	0.009	4.22
**16**	7	22.5	70	8	50.13	0.49	43.35	0.61	0.007	19.67
**17**	14	22.5	70	8	77.92	0.57	91.92	12.95	0.006	11.46
